# Data-driven assessment of corrosion in reinforced concrete structures embedded in clay dominated soils

**DOI:** 10.1038/s41598-025-08526-w

**Published:** 2025-07-02

**Authors:** Shahbaz Ahmad, Siraj Ahmad, Sabih Akhtar, Faraz Ahmad, Mujib Ahmad Ansari

**Affiliations:** 1 Geomechanics & Geotechnics, University of Kiel, Kiel, Germany; 2 Department of Civil Engineering, Aligarh Muslim University, Aligarh, India; 3 Narmada Hydroelectric Development Corporation Ltd., Bhopal, India; 4 Kiewit Inc., Denver, USA

**Keywords:** Corrosion behavior prediction, Cementitious composite materials, Neural network modeling, Reinforced steel durability, Engineering, Civil engineering

## Abstract

The integration of Artificial Intelligence techniques, particularly Artificial Neural Networks (ANNs), has transformed predictive modeling in structural and durability engineering. This study investigates the use of ANN-based approaches to predict the corrosion rates of mild steel reinforcement embedded in cementitious composites subjected to clay-dominated soil environments. Key environmental parameters, sodium chloride (NaCl) content (0-4%), inhibitor dosage (DOI) (0-5%), and exposure duration (30-180 days), were selected as input variables. Two ANN architectures, Feedforward Backpropagation (FFBP) and Cascadeforward Backpropagation (CFBP), were developed and trained using 72 experimental data points extracted from the literature. The FFBP model outperformed CFBP in terms of predictive accuracy, achieving a correlation coefficient (R) of 0.998, a mean absolute percentage error (MAPE) of 30.43%, and a root mean square error (RMSE) of 0.071 during testing. Sensitivity analysis revealed that inhibitor dosage had the most significant influence on corrosion behavior, followed by NaCl concentration and exposure duration. The findings confirm that ANN models can effectively capture the nonlinear interactions governing corrosion progression, even under complex environmental conditions associated with clayey soils. This research provides a reliable and practical AI-driven framework for assessing corrosion risk, guiding material design, and enhancing long-term infrastructure durability in aggressive subsurface conditions. The study underscores the growing relevance of machine learning in simulating time-dependent deterioration processes in geotechnical and structural materials.

## Introduction

Concrete has long served as the backbone of modern infrastructure, enabling the construction of buildings, highways, dams, and underground systems that shape cities and support economies^1^. Much of this infrastructure was built during the industrial and urban expansion of the last half-century^2^, with design lives of 50 to 100 years. As these structures age, maintenance and rehabilitation become critical, particularly in aggressive environments that accelerate deterioration. Among such environments, clay-dominated soils present unique durability challenges due to their high moisture retention, low permeability, high ion exchange capacity, and often acidic pH. These geochemical characteristics intensify the electrochemical processes that drive steel corrosion in buried reinforced concrete. Studies have shown that clay-rich media accelerate chloride ingress and corrosion initiation^3–6^, with significant implications for underground material performance^7–9^. Understanding and predicting corrosion behavior in such settings is vital for extending the service life of reinforced concrete structures. Concrete durability modeling has evolved through three major scientific paradigms: empiricism, theory, and computation^10^. Empirical studies established early understanding through observation, while theoretical models provided frameworks for predicting complex behaviors. Computational methods, including finite element modeling (FEM), later introduced more refined simulations. Alkam^11^ predicted service life for RC structures in chloride environments, while Lin and Xiang^12^ developed a model incorporating environmental and material parameters. Ahmad^13^ reviewed corrosion monitoring methods and predictive models. Classic models by Bazant^14^, Morinaga^15^, and Wang and Zhao^16^ described corrosion-induced cracking through mechanical-expansion models and FEM. These works form the foundation for durability prediction. Finite element-based numerical models have evolved significantly, incorporating coupled processes such as heat, moisture, and ion transport to simulate chloride diffusion and corrosion-induced damage^17^. Although these models provide mechanistic insight, their reliance on material-specific parameters and complex boundary conditions limits their scalability.

**Fig. 1:**
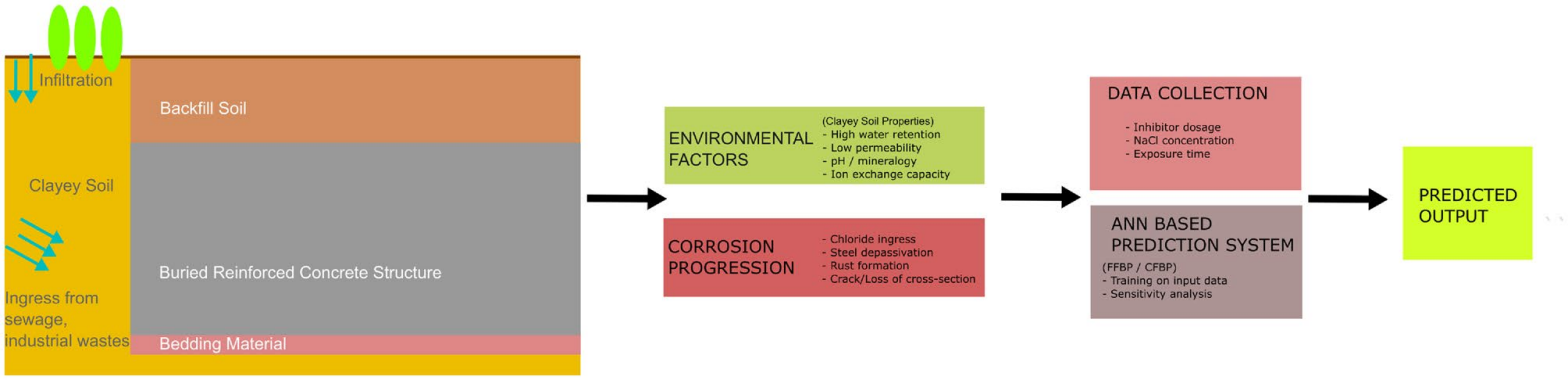
Conceptual workflow showing corrosion progression of a buried reinforced concrete structure in clayey soil, key environmental inputs, and the artificial neural network (ANN) framework used for corrosion rate prediction.

Moreover, such models are computationally intensive and less adaptable to large datasets arising from modern field monitoring campaigns, especially in geotechnical contexts involving expansive or problematic soils common in rural infrastructure^18^. To address these limitations, researchers are increasingly turning to artificial intelligence (AI) and machine learning (ML) approaches. Among them, artificial neural networks (ANNs) have gained prominence due to their capacity to learn nonlinear relationships from data, making them ideal for problems involving complex environmental interactions. Although ANNs have been in use for several decades, their continued relevance lies in their adaptability, transparency in architecture, and interpretability. Compared to more recent deep learning models such as convolutional neural networks (CNNs) and recurrent neural networks (RNNs), which are better suited for image processing and sequential time-series data respectively, shallow ANNs like FFBP and CFBP are computationally efficient, easier to train on smaller datasets, and highly effective for tabular experimental data with limited dimensionsas is the case in this study. Thus, ANN remains a relevant and practical modeling framework, particularly when explainability and fast convergence are important. AI has been extensively applied in civil engineering to predict material properties such as compressive strength^19,20^, crack propagation^21–24^, flexural and tensile strength^25–28^, shear capacity^29,30^, elastic modulus^31^, shrinkage^32,33^, and chloride diffusion^34,35^. Despite these advances, the direct application of ANNs to predict corrosion behavior in aggressive soil environments remains relatively limited^36^. However, recent studies have shown promise. Dong et al.^37^ applied ML to model steel corrosion embedded in soil, while Hosseinzadeh et al.^38^ predicted chloride resistance in concrete using AI. Song et al.^39^ explored interpretable ML for corrosion depth analysis, and Ji et al.^40^ applied recurrent neural networks for time-series corrosion forecasting. These developments reflect the growing relevance of AI-based models for durability assessments. While these contributions have improved mechanistic understanding, the integration of such physical models with data-driven approaches remains a significant research challenge^41^. Artificial intelligence, particularly ANNs, offers a paradigm shift by providing data-driven alternatives to conventional prediction models. These tools excel in scenarios with complex parameter interactions and incomplete mechanistic understanding. Furthermore, ANNs are adaptable, capable of being retrained or fine-tuned as new data becomes available, which makes them ideal for infrastructure monitoring and risk assessment frameworks. By demonstrating the feasibility and benefits of ANN-based corrosion modeling in clayey soils, the work contributes to a growing body of literature advocating for hybridized, intelligent infrastructure systems, (shown in Figure 1). To further explore this potential, the present study evaluates two ANN architectures, Feedforward Backpropagation (FFBP) and Cascadeforward Backpropagation (CFBP) for predicting corrosion rates of mild steel reinforcement in cementitious composites exposed to clay-rich soils. Input parameters include sodium chloride (NaCl) concentration, inhibitor dosage (DOI), and exposure duration (t). Literature sourced experimental data are used for model training and testing. Performance metrics such as mean absolute percentage error (MAPE) and coefficient of determination (R ) are employed to evaluate model accuracy. Additionally, sensitivity analysis is conducted to identify the relative influence of each input parameter. The overarching goal is not only to improve predictive capability but also to provide actionable insights for material design and durability planning. By identifying the dominant factors that influence corrosion in clayey environments, this study supports more informed engineering decisions regarding the selection of inhibitors, exposure thresholds, and material formulations. These insights can directly contribute to extending the service life of infrastructure and reducing lifecycle maintenance costs. Beyond its technical scope, this work addresses a broader research need: bridging the gap between classical mechanistic models and emerging data-driven tools. As the volume of field and lab generated durability data grows, the integration of AI into predictive modeling will become increasingly essential. In this context, corrosion prediction represents a frontier where data, materials science, and machine learning converge. This study represents a step toward that integration. By demonstrating the feasibility and benefits of ANN-based corrosion modeling in clayey soils, the work contributes to a growing body of literature advocating for hybridized, intelligent infrastructure systems. As we move toward smarter cities and more resilient construction practices, embedding predictive intelligence within materials research is not just an advantage, it is a necessity.

## Methods

### Formulation of neural network model and data

In order to map the relationship related to the rate of corrosion, an input-output scheme was used. From review of literature, it was concluded that the rate of corrosion depends upon: (i) Salinity level (NaCl), (ii) Dose of inhibitor (DOI), (iii) Exposure duration (t). The model thus takes the input in the form of causative factors namely NaCl, DOI and t and yields the output as Corrosion rate (CR).

1 $$\begin{aligned} CR = f(\text {NaCl}, \text {DOI}, t) \end{aligned}$$

The input and output variables involved in the present ANN model were first normalized within the range of 0 to 1 as follows:

2 $$\begin{aligned} X_N = \frac{X - X_{\text {min}}}{X_{\text {max}} - X_{\text {min}}} \end{aligned}$$

where $X_N$ is the normalized value of *X*, and $X_{\text {max}}$ and $X_{\text {min}}$ are the maximum and minimum values of each variable. This normalization allowed the network to be trained more effectively.

The dataset used in this study to train and validate the ANN models was derived from the experimental work of Akhtar et al.^42^, which investigated the corrosion behavior of reinforced concrete samples under controlled laboratory conditions. The corrosion rate data were generated by systematically varying three primary input parameters: sodium chloride (NaCl) concentration, corrosion inhibitor dosage (DOI), and exposure duration (t). These variables were selected due to their well-established influence on electrochemical corrosion processes affecting steel embedded in cementitious environments. The dataset comprises 72 distinct experimental observations, encompassing a representative range of conditions: NaCl concentrations from 0% to 4%, inhibitor dosages from 0% to 5%, and exposure durations between 30 and 180 days. The corrosion rate (CR) values, reported in mils per year (mpy), were derived from gravimetric mass loss testing as described in Akhtar et al.^42^. To prepare the data for model training, all input and output variables were normalized to a [0, 1] scale using min-max normalization to ensure uniform scaling, reduce bias due to magnitude differences, and improve training stability. Subsequently, the dataset was randomly partitioned into training, validation, and testing subsets using an 80:10:10 split. The training set (80%) was used to derive the model, while the remaining 20% of the data, unseen during training was reserved for validation and testing to ensure unbiased performance evaluation.

**Fig. 2:**
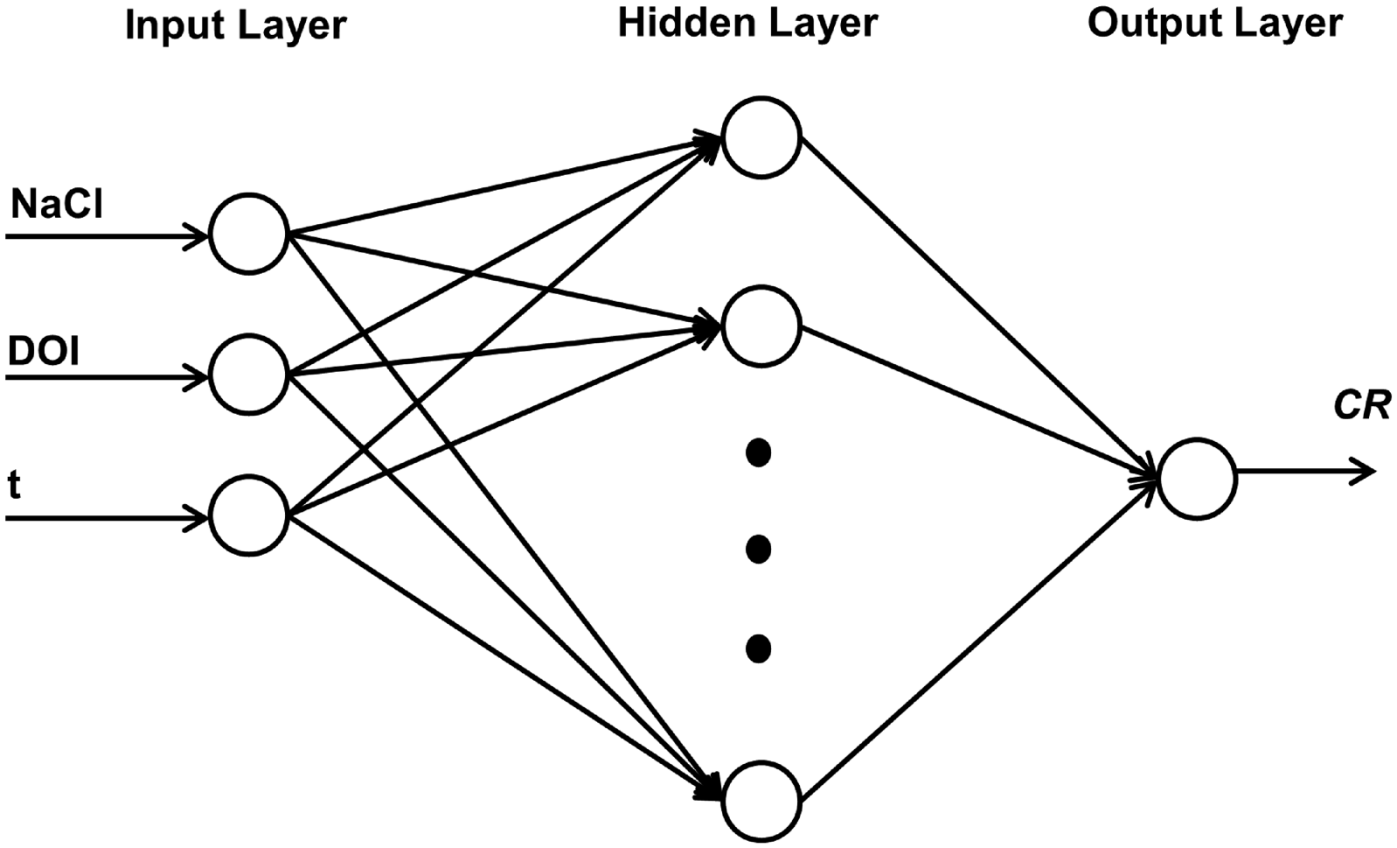
Architecture of present ANN model.

In the present work, different types of networks were considered and trained using a back-propagation algorithm. The resulting neural network models are referred to as Feedforward Backpropagation (FFBP) and Cascadeforward Backpropagation (CFBP). In this study, ANN models with a single hidden layer were developed. Identifying the number of neurons in the input and output layers is straightforward, as it is determined by the input and output variables considered in the physical process model. However, determining the optimal number of hidden layer nodes required a trial-and-error approach to identify the best network configuration. The optimal architecture was determined by varying the number of hidden neurons, aiming to minimize the difference between the predicted values from the neural network model and the desired output. Generally, as the number of neurons in the hidden layer increases, the network’s prediction capability improves initially and then stabilizes. For training, a gradient descent algorithm was employed, with the number of training epochs set to 1000. The performance of all neural network model configurations was evaluated based on the coefficient of correlation (*R*) between the predicted values and the desired output, Mean Absolute Percentage Error (MAPE), and Root Mean Square Error (RMSE). The training was stopped when either an acceptable level of error was achieved or when the maximum number of iterations was reached. The neural network model configuration that minimized MAPE and optimized *R* was selected and the entire analysis was repeated several times. The architecture of the present ANN model is shown in Figure 2. The dataset used in this study, shown in Table 1^42^, highlights the progressive increase in corrosion rate (CR) over time under conditions with no sodium chloride (NaCl) or inhibitor dosage (DOI). This data provides critical insights into the time-dependent behavior of corrosion, forming the basis for further analysis and modeling. The determination of the network configuration, including the optimal number of hidden nodes, was a critical step in this study. While the number of input and output nodes ($I = 3$ and $O = 1$, respectively) was straightforward, as dictated by the causative factors and output variable, optimizing the number of hidden layer neurons required an iterative trial-and-error process. Various configurations were tested to achieve a balance between minimizing error metrics–such as Mean Absolute Percentage Error (MAPE) and Root Mean Square Error (RMSE)and maximizing the coefficient of correlation ($R$). The optimal configurations identified for the Feedforward Backpropagation (FFBP) and Cascadeforward Backpropagation (CFBP) models were 20 and 22 hidden neurons, respectively, as summarized in Table 2. Additionally, the learning rate and momentum factor were set at 0.5 and 0.7, respectively, to ensure stable and efficient convergence during training. Training was stopped when the error reached a predefined threshold of 0.0001 or when 1000 epochs were completed. This systematic optimization process ensured the development of robust and accurate ANN models for corrosion prediction. All ANN models, including FFBP and CFBP architectures, were implemented using the Neural Network Toolbox in MATLAB (MathWorks, Natick, MA, USA)^43^.

**Table 1:** Data selected for this study^42^.

S.No.	NaCl (%)	DOI (%)	t (days)	CR (mpy)
1	0	0	30	1.166
2	0	0	60	1.193
3	0	0	90	1.219
4	0	0	120	1.303
5	0	0	150	1.388
6	0	0	180	1.473
7	0	1	30	0.091
8	0	1	60	0.097
9	0	1	90	0.104
10	0	1	120	0.113
11	0	1	150	0.122
12	0	1	180	0.131
13	0	3	30	0.039
14	0	3	60	0.039
15	0	3	90	0.065
16	0	3	120	0.071
17	0	3	150	0.076
18	0	3	180	0.082
19	0	5	30	0.082
20	0	5	60	0.036
21	0	5	90	0.047
22	0	5	120	0.049
23	0	5	150	0.052
24	0	5	180	0.054
25	2	0	30	1.443
26	2	0	60	1.498
27	2	0	90	1.553
28	2	0	120	2.000
29	2	0	150	2.414
30	2	0	180	2.920
31	2	1	30	0.111
32	2	1	60	0.120
33	2	1	90	0.201
34	2	1	120	0.440
35	2	1	150	0.675
36	2	1	180	0.835
37	2	3	30	0.600
38	2	3	60	0.064
39	2	3	90	0.063
40	2	3	120	0.080
41	2	3	150	0.105
42	2	3	180	0.145
43	2	5	30	0.013
44	2	5	60	0.024
45	2	5	90	0.035
46	2	5	120	0.058
47	2	5	150	0.085
48	2	5	180	0.118
49	4	0	30	1.581
50	4	0	60	1.651
51	4	0	90	1.721
52	4	0	120	2.362
53	4	0	150	3.002
54	4	0	180	3.643
55	4	1	30	0.121
56	4	1	60	0.177
57	4	1	90	0.232
58	4	1	120	0.470
59	4	1	150	0.710
60	4	1	180	0.947
61	4	3	30	0.069
62	4	3	60	0.075
63	4	3	90	0.088
64	4	3	120	0.100
65	4	3	150	0.130
66	4	3	180	0.160
67	4	5	30	0.016
68	4	5	60	0.280
69	4	5	90	0.040
70	4	5	120	0.067
71	4	5	150	0.097
72	4	5	180	0.122

### Soil type consideration in corrosion modeling

The experimental dataset used in this study was obtained under controlled laboratory conditions, varying sodium chloride (NaCl) concentration, inhibitor dosage (DOI), and exposure time (t); the findings have direct relevance to clay-dominated soil environments. Clayey soils, characterized by their fine-grained texture, high water retention, low permeability, and ion exchange capacity, create conditions that significantly accelerate reinforcement corrosion^3–6^. These soils facilitate the accumulation of aggressive ions such as chlorides at the steel concrete interface while impeding oxygen diffusion, leading to early depassivation and rapid corrosion progression. The experimental data were obtained from the work of Akhtar et al.^42^, in which mild steel reinforcement bars embedded in cementitious specimens were exposed to sodium nitrite-based inhibitors under simulated clayey soil conditions. The testing protocol varied NaCl (0-4%), inhibitor dosage (0-5%), and exposure time (30-180 days) under controlled laboratory settings. Corrosion progression was monitored using the gravimetric mass loss method, providing a consistent and high-fidelity dataset for ANN training.

While this study focuses broadly on clay-rich environments, it is important to acknowledge the mineralogical variability within the clay category, which can significantly influence corrosion behavior. Different clay minerals, such as smectite, kaolinite, and illite, exhibit distinct physicochemical characteristics, including cation exchange capacity (CEC), swelling potential, and permeability. Smectite-rich soils, for instance, retain more moisture and ions due to their high CEC and expansive nature, creating a more aggressive electrochemical environment for steel^5,44^. In contrast, kaolinite exhibits lower reactivity and allows greater oxygen diffusion, resulting in different corrosion dynamics^45^. Additionally, Illite presents intermediate behavior but can significantly impact corrosion under variable pH or chloride conditions^44^. Although the ANN model developed in this study does not differentiate between clay mineral types, its ability to capture the effects of key environmental variables: NaCl content, inhibitor dosage, and exposure time, offers a valuable step toward real-world corrosion assessment. These factors, which govern electrochemical conditions in diverse clay environments, are critical for understanding the long-term durability of buried reinforced concrete structures, including foundations, retaining walls, tunnels, and pipelines. The developed ANN models provide a practical and computationally efficient framework for predicting corrosion risks in clay-rich geotechnical settings. This approach enhances the field applicability of AI-driven corrosion modeling, bridging the gap between laboratory experimentation and infrastructure durability assessment under variable site conditions.

### Model performance evaluation

The performance of all the models of ANN and SVM configurations is evaluated based on various metrics such as the coefficient of correlation ($R$), coefficient of determination (R ), Nash-Sutcliffe efficiency coefficient ($E$), Root Mean Squared Error (RMSE), Mean Absolute Percentage Error (MAPE), Absolute Percentage Error (APE), Average Absolute Deviation (AAD), and Scatter Index (SI). These metrics offer a comprehensive and versatile framework for assessing the performance of ANN and SVM configurations in capturing and predicting intricate phenomena. By evaluating both overall predictive accuracy and instance-level performance, this methodology facilitates a detailed and dependable comparison of the models. Furthermore, the use of these metrics promotes clarity and reproducibility, enabling a robust analysis of the strengths and limitations inherent in each configuration. These metrics are defined as follows:

**1. Coefficient of Correlation (R** **) and Coefficient of Determination (R** ^**2**^ **)**

The coefficient of correlation ($R$) describes the degree of collinearity between simulated and observed data, ranging from -1 to 1. A perfect positive or negative linear relationship exists if $R = 1$ or $R = -1$, while $R = 0$ indicates no linear relationship. It is calculated as:

3 $$\begin{aligned} R = \frac{\sum _{i=1}^{n}(O_i - \overline{O})(P_i - \overline{P})}{\sqrt{\sum _{i=1}^{n}(O_i - \overline{O})^2 \sum _{i=1}^{n}(P_i - \overline{P})^2}} \end{aligned}$$

Here, $O_i$ and $P_i$ are the observed and predicted values, while $\overline{O}$ and $\overline{P}$ are their respective means.

**2. Nash-Sutcliffe Efficiency Coefficient (E)**

The Nash-Sutcliffe efficiency coefficient ($E$) assesses the predictive power of models. It is defined as:

4 $$\begin{aligned} E = 1 - \frac{\sum _{i=1}^{n}(O_i - P_i)^2}{\sum _{i=1}^{n}(O_i - \overline{O})^2} \end{aligned}$$

$E = 1$ indicates a perfect match, $E = 0$ suggests the model is as accurate as the mean of the observed data, and $E < 0$ reflects unacceptable performance.

**3. Root Mean Squared Error (RMSE)**

RMSE is a measure of the difference between values predicted by a model and those observed. It is given by:

5 $$\begin{aligned} RMSE = \sqrt{\frac{\sum _{i=1}^{n}(O_i - P_i)^2}{n}} \end{aligned}$$

**4. Mean Absolute Percentage Error (MAPE)**

MAPE expresses accuracy as a percentage and is defined as:

6 $$\begin{aligned} MAPE = \frac{1}{n} \sum _{i=1}^{n} \left| \frac{O_i - P_i}{O_i} \right| \times 100 \end{aligned}$$

**5. Absolute Percentage Error (APE)**

The absolute percentage error is calculated as:

7 $$\begin{aligned} APE = \left| \frac{O_i - P_i}{O_i} \right| \times 100 \end{aligned}$$

**6. Average Absolute Deviation (AAD)**

The average absolute deviation measures statistical dispersion and is given by:

8 $$\begin{aligned} AAD = \frac{\sum _{i=1}^{n} |O_i - P_i|}{n} \end{aligned}$$

**7. Scatter Index (SI)**

The scatter index is a normalized measure of the scatter of data points and is defined as:

9 $$\begin{aligned} SI = \frac{RMSE}{\overline{O}} \end{aligned}$$

**8. Standard Deviation Absolute Percentage Error (SDAPE)**

The SDAPE evaluates the deviation of absolute percentage errors and is expressed as:

10 $$\begin{aligned} SDAPE = \sqrt{\frac{1}{n} \sum _{i=1}^{n} \left( \left| \frac{O_i - P_i}{O_i} \right| - MAPE \right) ^2} \end{aligned}$$

## Results and discussion

In this section, the analysis of data related to the prediction of corrosion rates in cementitious composites is presented. A generalized neural network model has been developed to predict corrosion rates with high accuracy. Additionally, a sensitivity analysis was performed to evaluate the relative importance of each independent parameter (input neurons) in influencing the model’s predictions. This approach provides insights into the key factors driving corrosion and enhances the reliability of the predictive model.

### Numerical results of artificial neural network model

All patterns were normalized within the range of 0.0 to 1.0 before their use. Similarly, all weights and bias values were initialized to random numbers. While the numbers of input and output nodes were fixed, the hidden nodes were subjected to trials, and the configuration producing the most accurate result in terms of the correlation coefficient was selected.

### Training and error evaluation

**Fig. 3:**
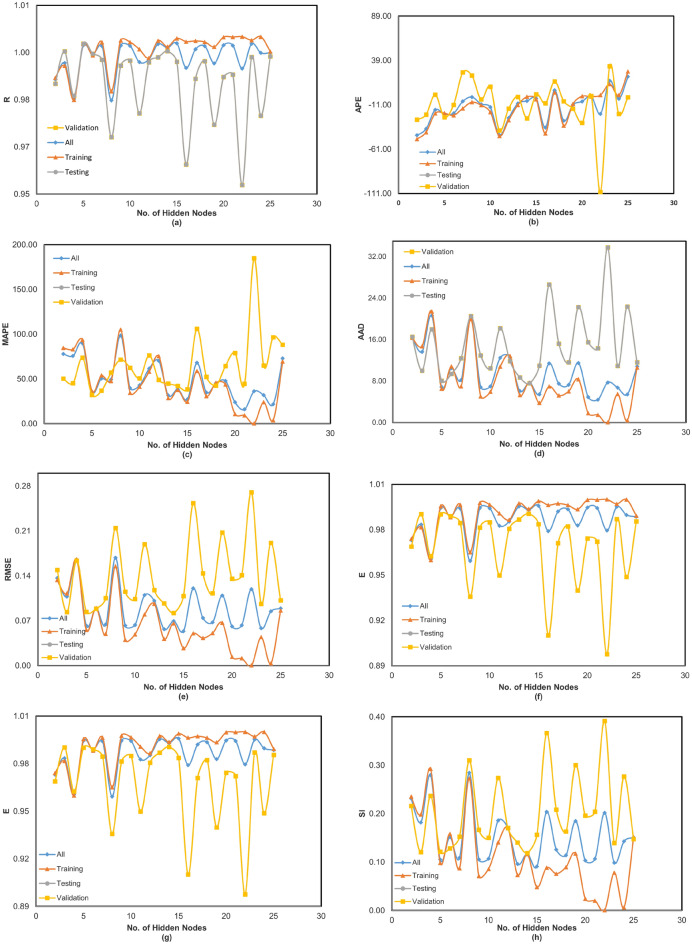
(**a-h**) Variation of performance parameters with number of hidden neurons in the CFBP model. “Training,” “Validation,” and “Testing” correspond to the respective subsets of the 80:10:10 data split. “All” represents the model’s performance across the full dataset, included for visualization purposes.

**Fig. 4:**
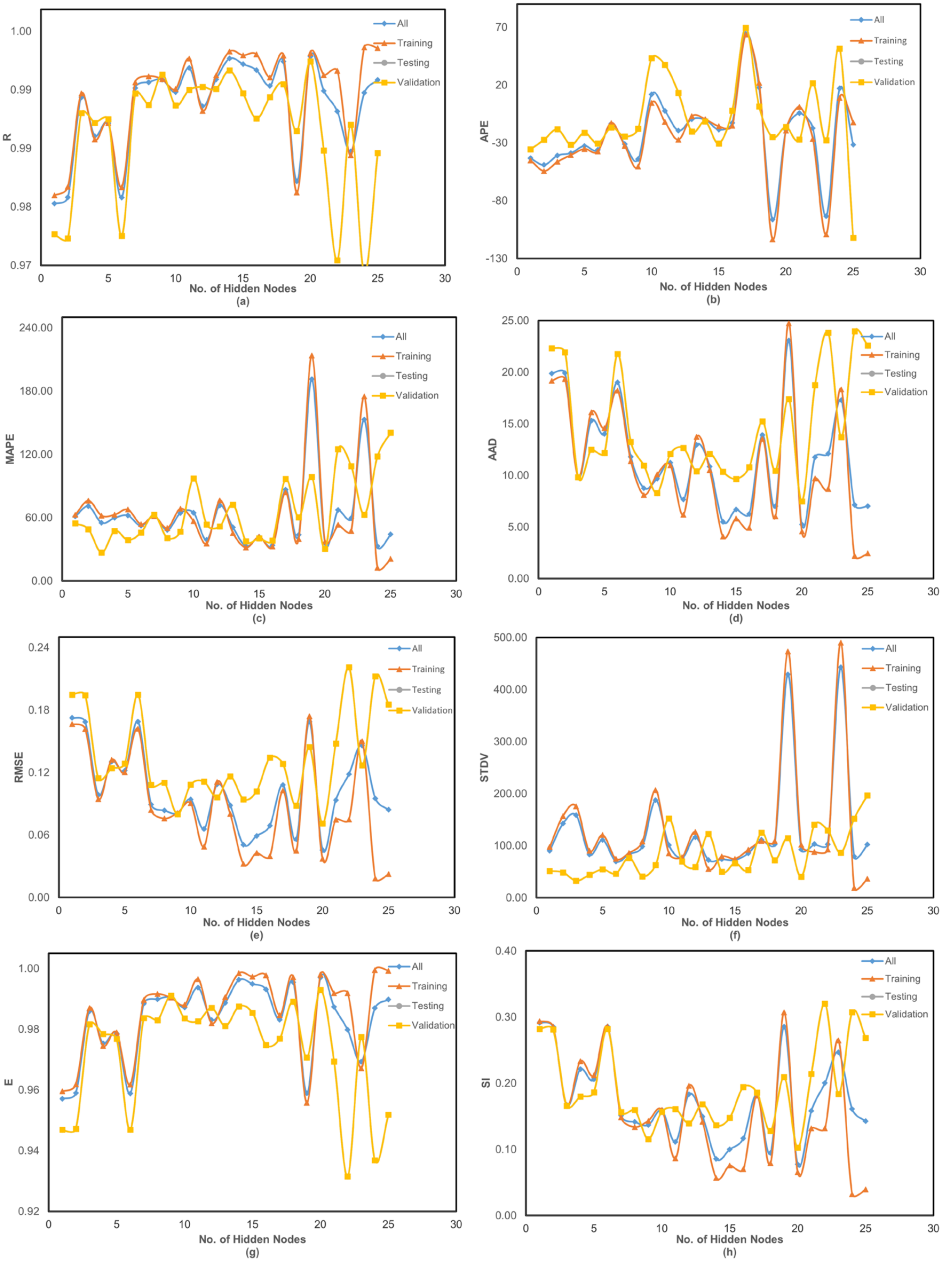
(**a-h**) Variation of performance parameters with number of hidden neurons in the FFBP model. “Training,” “Validation,” and “Testing” correspond to the respective subsets of the 80:10:10 data split. “All” represents the model’s performance across the full dataset, included for visualization purposes.

**Fig. 5:**
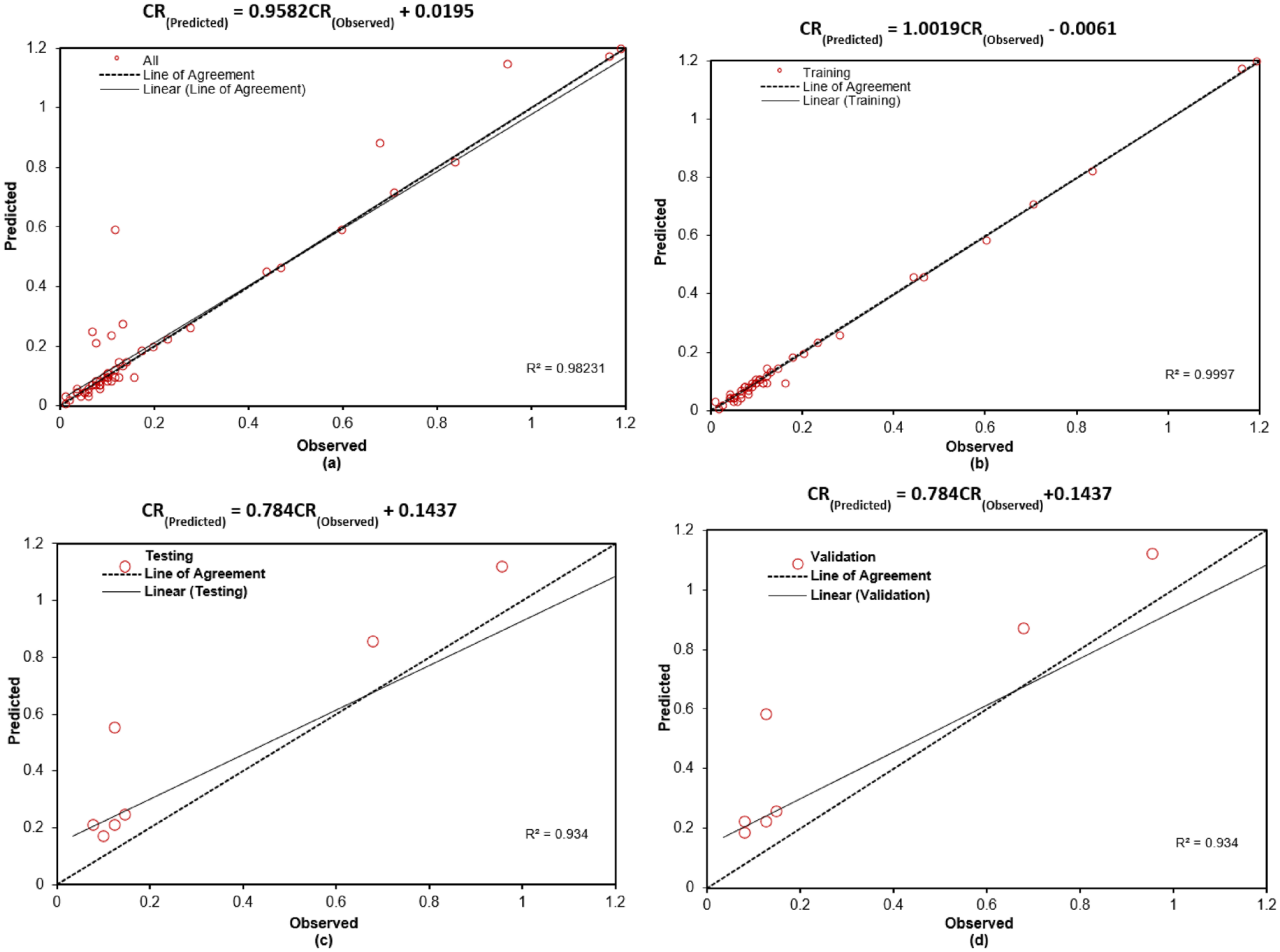
(**a-d**) Comparison between observed and predicted values of CR by CFBP.

**Fig. 6:**
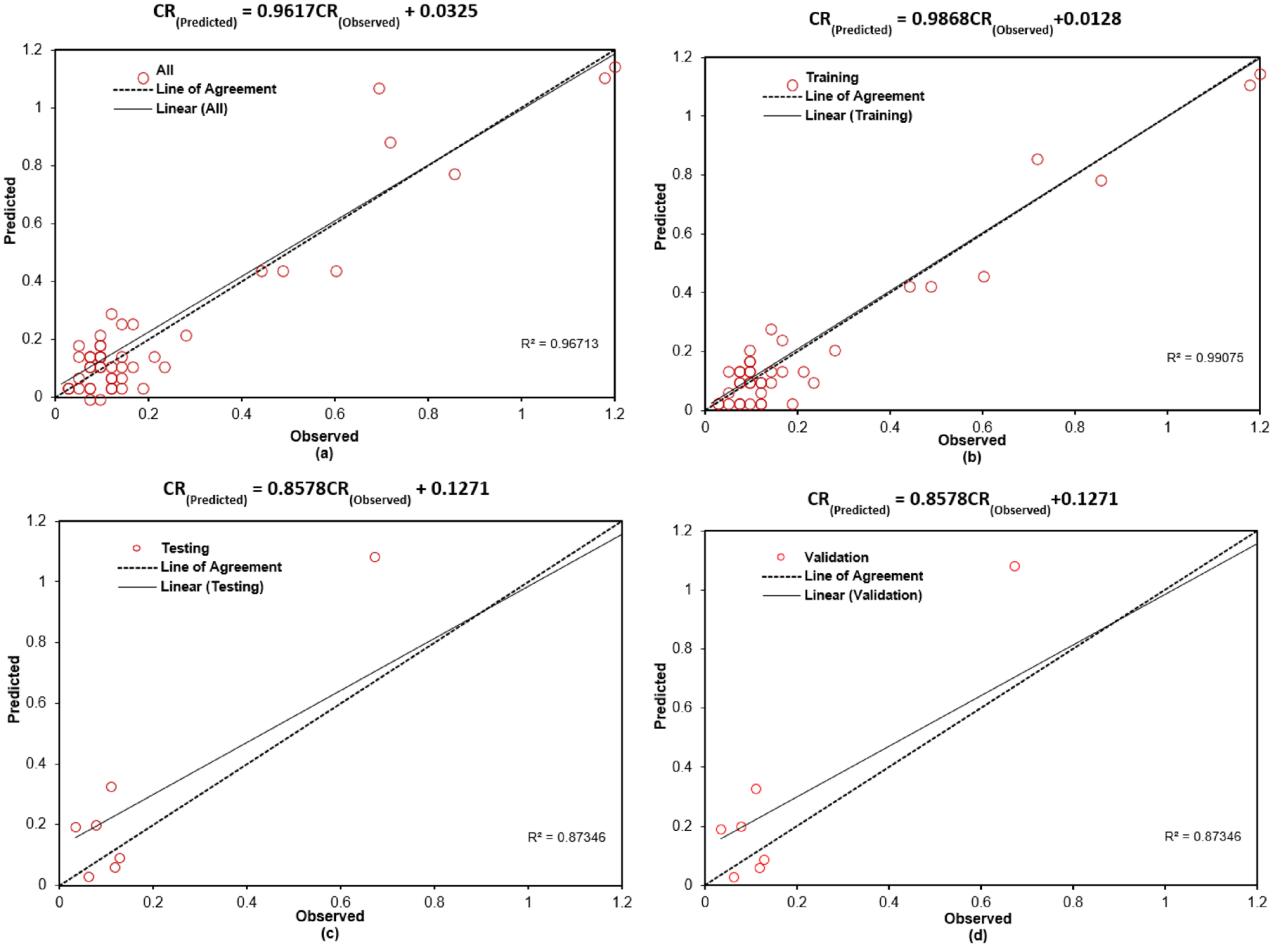
(**a-d**) Comparison between observed and predicted values of CR by FFBP.

Figures 3 and 4 illustrate the variation of error as a function of the number of hidden nodes for the present ANN models. Specifically, Figure 3(a) to (h) correspond to the Cascadeforward Backpropagation (CFBP) model, while Figures 4(a) to 4(h) pertain to the Feedforward Backpropagation (FFBP) model. The training of each neural network model was terminated once the mean squared error (MSE) between the network output and the actual output for all training data reached the predefined minimum threshold of 0.0001. This ensured that the models achieved optimal training convergence without overfitting. To evaluate the performance of the ANN models, multiple error estimation metrics coefficient of correlation ($R$), coefficient of determination (R ), mean absolute percentage error (MAPE), root mean square error (RMSE), Nash-Sutcliffe Efficiency Coefficient ($E$), and Scatter Index (SI) were computed. These performance metrics are summarized in Table 3 for the network configurations specified in Table 2. Additionally, the network architecture derived from equation (1) is shown in Figure 2, while the trained weights and bias values for different ANN models are listed in Tables 4 and 5. The predicted corrosion rates are shown in Figures 5 and 6, with the observed values for all ANN models, providing a visual assessment of their predictive accuracy.

Notably, while the results for non-normalized data are not included, it was observed that normalization significantly enhanced the training process by improving the network’s convergence and accuracy. A detailed analysis of Table 3 and Figures 5 and 6 reveals that, when considering all error criteria collectively, the FFBP model outperforms the CFBP model in terms of overall accuracy for predicting corrosion rates. This difference can be attributed to the CFBP model’s greater architectural complexity, which may lead to overfitting on smaller datasets. The FFBP model, with its simpler structure, demonstrated more stable performance and better generalization across all subsets. Although the FFBP model demonstrated better results (Table 4), the connection weights and biases for the CFBP architecture are also provided in Table 5 for completeness and potential reference in future comparative studies.

The performance of the developed ANN models in this study demonstrates a notable improvement over existing models reported in the literature. Few previous studies have achieved correlation coefficients R ranging from 0.55 to 0.61 with mean absolute percentage errors MAPE between 39% and 53% when predicting corrosion depths in steel and zinc under atmospheric conditions^46^. Another study focusing on corrosion current density prediction in reinforced concrete by Nikoo et al.^47^ employed a self-organizing feature map (SOFM) optimized with a genetic algorithm to predict corrosion current density in reinforced concrete, achieving an R of 0.978 and RMSE of 0.02 during the testing phase. In contrast, our ANN model achieved an R of 0.999 with significantly lower RMSE and MAPE values, indicating superior predictive accuracy. Moreover, while prior models often utilized diverse and less controlled datasets, our approach benefits from a well-structured and consistent dataset derived from controlled laboratory experiments, enhancing the model’s reliability. To our knowledge, this study is among the first to apply ANN techniques specifically to predict corrosion rates in clay-rich geotechnical environments, addressing a niche yet critical area in infrastructure durability assessment.

The FFBP model achieved the highest coefficient of correlation (R) and the lowest MAPE and RMSE values. During training, the FFBP model recorded R = 0.999, MAPE = 37.12%, and RMSE = 0.037. During testing, it achieved R= 0.998, MAPE = 30.43%, and RMSE = 0.071. While the MAPE appears higher than RMSE or R, this is largely due to the wide range of corrosion rates in the dataset (0.014 3.60 mpy). As a percentage-based metric, MAPE is sensitive to small actual values, where even minor absolute errors can yield disproportionately large percentage deviations. This limitation is common in skewed datasets; therefore, MAPE is interpreted in conjunction with RMSE and R, which together confirm the model’s strong predictive performance. Overall, all models exhibited small MAPE, RMSE, and SI values during training, indicating reliable performance. Slightly higher error values were observed during validation, reflecting the inherent variability in unseen data. Despite this, the ANN models maintained consistently high correlation during testing, underscoring their robustness. To summarize, the network configuration of the FFBP model, along with the corresponding weights and bias matrices provided in Table 4, is recommended for general use in predicting corrosion rates. Its superior performance across all error metrics establishes it as the reliable model for practical applications.

**Table 2:** Network Architecture of the Present ANN Model.

Model	Algorithm	Network Configuration			Learning Rate	Momentum Function
Model-M	FFBP	I	H	O	0.5	0.7
		3	20	1		
	CFBP	3	22	1	0.5	0.7

$I$, $H$, and $O$ indicate the number of input, hidden, and output nodes, respectively

**Table 3:** Comparison between FFBP and CFBP model.

Model		R	APE	MAPE	AAD	RMSE	SDAPE	E	SI
FFBP	All	0.9985	-18.8322	35.8170	5.2332	0.0456	92.7299	0.9970	0.0771
	Training	0.9990	-19.4563	37.1181	4.5743	0.0369	101.6642	0.9980	0.0652
	Testing	0.9978	-16.2467	30.4268	7.4738	0.0709	40.0791	0.9929	0.1026
CFBP	All	0.9898	-21.2641	36.1292	7.7083	0.1192	111.6837	0.9795	0.2016
	Training	1.0000	-0.0127	0.2383	0.0441	0.0003	0.4322	1.0000	0.0006
	Testing	0.9528	-109.305	184.820	33.7668	0.2703	196.3811	0.8976	0.3913

**Table 4:** Connection Weights and Biases for FFBP MODEL.

No. of Neuron	Out Bias = 1.089922			Output Weight	Input Biases
	Input Weight				
	a	b	c		
1	1.21263	0.78591	-0.39795	-0.407179	-4.93189
2	0.57618	-1.12909	-2.62909	0.136300	-3.62555
3	0.99303	0.06761	2.68523	0.049079	-3.35861
4	2.36508	-2.47028	0.82848	-0.019910	-3.17869
5	-2.61617	2.06859	1.51980	-0.065128	1.01977
6	1.85537	-0.14366	-3.18980	-0.069800	-1.78938
7	2.89561	0.41687	0.29215	-0.332588	-1.56789
8	-3.51667	1.78554	-2.55521	-0.421287	-1.31408
9	1.53687	1.66653	2.93010	0.236246	0.296837
10	-4.83446	-0.92977	0.68700	-0.929879	-0.78849
11	-1.26126	-1.37225	1.33589	0.781673	-1.41168
12	-1.38164	-3.26542	-1.19647	0.167012	2.036181
13	1.65825	3.83562	-1.64534	0.102414	-0.40581
14	3.83193	-2.02473	-0.52001	0.228672	0.698213
15	-1.97681	2.25256	-1.95889	0.422880	-2.41621
16	2.31644	0.44307	2.85107	-0.123003	2.28434
17	-1.27856	3.27099	2.51386	0.014693	-1.1594
18	-3.66928	0.47191	-0.79165	0.955364	-3.36605
19	-2.55487	-0.16069	0.16859	-0.896383	-3.65256
20	0.14901	-2.86487	0.17576	2.036393	-3.37994
21	-0.99909	-3.28899	-0.3512	-0.358014	-3.022368
22	2.459115	-1.13785	0	0.401266	4.179342

**Table 5:** Connection Weights and Biases for CFBP MODEL.

No. of Neuron	Out Bias = 1.062898			Output Weight	Input Biases
	Input Weight				
	a	b	c		
1	0.265803	-1.96283	2.469871	-0.078765	-3.864874
2	2.121904	2.166686	1.014074	0.738640	-3.802204
3	2.676152	0.789435	1.079279	0.670242	-2.787769
4	-1.39779	1.88203	-1.95332	-0.268880	2.187476
5	0.499855	-2.77354	1.67264	-0.279297	-2.076016
6	-0.82424	-1.16482	2.994811	0.046704	1.689123
7	-1.96833	-1.77352	-0.36325	1.363907	2.857356
8	-0.46512	3.211127	-0.55542	-0.015775	0.349130
9	-1.28996	-2.1446	0.909958	-0.408602	2.042473
10	2.278871	1.187861	-1.81018	-0.360981	-1.416722
11	-1.40878	2.027771	-2.25335	-0.175346	-0.112553
12	0.50845	2.000311	-0.74561	0.492385	-0.160822
13	-1.44873	-1.38192	-2.32382	-0.359848	-0.457329
14	1.707984	-0.82084	-2.85562	-0.205090	0.649073
15	-2.38538	-2.27714	1.464113	0.414439	-1.123444
16	0.822718	2.029048	-1.60294	-0.339372	2.495753
17	0.90201	-0.72755	3.029773	-0.108637	1.578084
18	1.562758	2.6311	2.404521	-0.183669	1.448191
19	0.274387	-2.88807	0.15367	1.799537	-3.203242
20	-3.4017	-1.36612	-0.19832	-0.195448	-2.541945
21	-0.99909	-3.28899	-0.3512	-0.358014	-3.022368
22	2.459115	-1.13785	0	0.401266	4.179342

### Sensitivity analysis

Sensitivity tests were conducted to ascertain the relative significance of each of the independent parameter (input neurons) on the corrosion rate given by equation 1. In the sensitivity analysis, each input neuron was in turn eliminated from the model, and its influence on the prediction of corrosion rate was evaluated in terms of the $\hbox {R}^2$, MAPE, RMSE, E, and SI criteria. The network architecture of the problem as shown in Table 2 considered in the present sensitivity analysis consist of a hidden layer with 20 neurons in layer for FFBP and 22 neurons in CFBP respectively. Comparison of different neural networks models with one of the independent parameter removed in each case is presented in Tables 6 and 7.

**Fig. 7:**
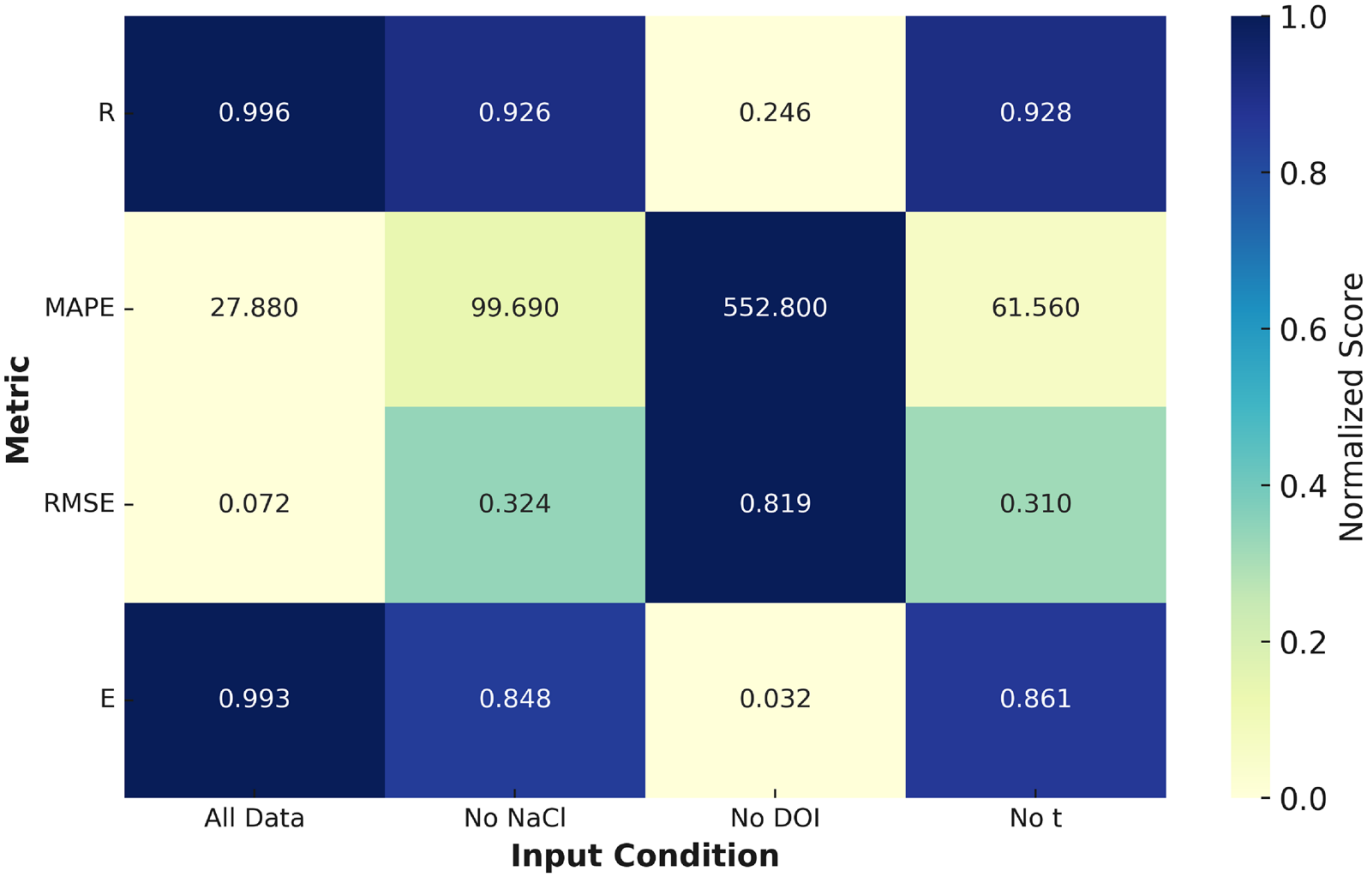
Refined sensitivity analysis heatmap of the FFBP model showing the effect of excluding input variables on predictive accuracy. The inhibitor dosage (DOI) is identified as the most critical input.

The results shown in Table 6 and Heat Map (Figure 7) presents that, for the most suitable model (FFBP), the inhibitor dosage (DOI) is the most significant input parameter for predicting the corrosion rate. This conclusion is supported by the sharp drop in the correlation coefficient (R) from 0.996 to 0.246 and the corresponding increase in MAPE from 27.88% to 552.8% when DOI is excluded from the model. This further indicates that the presence of corrosion inhibitor plays a dominant role in governing electrochemical behavior under the tested environmental conditions. The variables in order of decreasing level of sensitivity for FFBP model are: DOI, NaCl, t. These findings are consistent with recent literature on the relative importance of NaCl concentration, inhibitor dosage, and exposure time in influencing steel corrosion^42^. Sodium chloride promotes chloride-induced depassivation of steel and accelerates corrosion initiation^48^. Inhibitor dosage has been widely reported as a key controlling factor in mitigating corrosion through passive layer stabilization^49^. Exposure time governs the extent of electrochemical interaction and progressive deterioration under sustained conditions^50^.

The sensitivity analysis of the FFBP and CFBP models suggests that ’t’ have only a marginal influence on the resulting corrosion rate compared to the other parameters. However, considering the limitation and uncertainties in the data, a full-fledged network involving all input variables would be desirable. In view of the variability in the outcome resulting from application of different analytical schemes (ANNs models), it is felt that the network that requires all input quantities may be followed for generality.

**Table 6:** Sensitivity Analysis by FFBP.

	NNR	R	APE	MAPE	AAD	RMSE	STDV	E	SI	Performance
All DATA	20	0.996	5.213	27.88	6.185	0.072	50.34	0.993	0.121	All
		1	12.48	16.22	2.684	0.02	25.34	0.999	0.036	Training
		0.985	-24.88	76.21	18.09	0.157	88.89	0.965	0.228	Validation
NO NaCl	20	0.926	-81.09	99.69	32.63	0.324	167.4	0.848	0.548	All
		0.921	-84.45	104.5	33.81	0.333	183.1	0.838	0.587	Training
		0.945	-67.16	79.83	28.62	0.284	74.7	0.887	0.411	Validation
NO DOI	20	0.246	-518.8	552.8	117.5	0.819	611.4	0.032	1.386	All
		0.359	-514.4	546.3	117.1	0.78	626	0.113	1.375	Training
		-0.193	-537.4	580.1	119	0.966	568	-0.308	1.399	Validation
NO t	20	0.928	-39.35	61.56	29.87	0.31	95.16	0.861	0.525	All
		0.942	-40.92	61.97	27.1	0.277	102.3	0.888	0.489	Training
		0.872	-32.87	59.87	39.3	0.419	59.79	0.754	0.607	Validation

**Table 7:** Sensitivity Analysis by CFBP.

	NNR	R	APE	MAPE	AAD	RMSE	STDV	E	SI	Performance
All DATA	22	0.993	-15.38	37.13	7.046	0.098	91.68	0.986	0.167	All
		1	-12.55	17.88	2.189	0.017	42.73	1	0.03	Training
		0.971	-27.11	116.9	23.56	0.221	171.4	0.932	0.319	Validation
NO NaCl	22	0.932	-59.43	82.98	29.66	0.303	121.8	0.868	0.512	All
		0.944	-56.63	79.41	27.65	0.274	121.6	0.891	0.483	Training
		0.888	-71.01	97.78	36.51	0.4	126.2	0.776	0.579	Validation
NO DOI	22	0.238	-432.4	469.5	108	0.816	678.1	0.039	1.381	All
		0.338	-449.4	485	108.3	0.779	731.1	0.114	1.375	Training
		-0.141	-362.3	405.2	106.6	0.954	403.4	-0.276	1.381	Validation
NO t	22	0.928	-38.82	60.9	29.87	0.31	92.14	0.861	0.525	All
		0.942	-40.27	61.29	27.08	0.277	98.71	0.888	0.489	Training
		0.872	-32.81	59.29	39.38	0.42	60.38	0.753	0.608	Validation

### Practical applications

The proposed ANN models provide a robust computational tool for predicting corrosion rates in reinforced cementitious composites, with significant implications for real-world applications. By leveraging the ability of artificial neural networks to simulate complex interactions, these models can address several critical challenges in the design, maintenance, and sustainability of civil infrastructure.

1. **Design Optimization** The models can guide the design of durable concrete mixtures by optimizing the dosage of corrosion inhibitors based on site-specific environmental conditions, such as chloride exposure levels. This capability ensures that the design specifications are tailored to minimize corrosion risks, thereby enhancing the longevity of structures.
2. **Infrastructure Maintenance** Predictions generated by the models can inform infrastructure maintenance schedules, enabling proactive and timely interventions. For example, bridges, dams, and culverts exposed to saline environments are particularly vulnerable to corrosion. The ANN models can help prioritize these structures for inspection and remediation, reducing the likelihood of structural failures.
3. **Cost Reduction** Early predictions of high corrosion rates in specific environmental conditions allow for better material selection and preemptive adjustments during the design phase. This reduces long-term maintenance costs and the need for expensive retrofitting. Moreover, optimizing inhibitor dosage ensures efficient use of materials, minimizing waste and associated costs.
4. **Policy and Planning** Civil engineering firms and regulatory bodies can use the model’s outputs to establish guidelines and policies for chloride content limits, minimum inhibitor dosages, and maintenance standards. Such data-driven policies can improve overall infrastructure resilience while promoting sustainable development.
5. **Emergency Retrofitting** In critical situations where rapid corrosion poses an immediate risk, the models can identify high-priority structures requiring urgent retrofitting. This capability supports emergency response planning and helps prevent catastrophic failures by ensuring that resources are allocated effectively.
6. **Sustainability and Environmental Impact** By facilitating the design of corrosion-resistant materials and reducing unnecessary overdesign, the models contribute to more sustainable construction practices. This aligns with global goals to reduce the environmental footprint of infrastructure projects.

Future work may focus on incorporating additional variables such as pH, temperature, and moisture content to enhance prediction accuracy. Coupling the ANN framework with geospatial data could support corrosion risk mapping, while integrating probabilistic models would help quantify uncertainty. Expanding the dataset with multi-source experimental data may also enable more generalizable and hybrid AI modeling approaches.

## Conclusions

This study demonstrates the potential of artificial neural networks (ANNs) as effective computational tools for predicting corrosion rates in reinforced cementitious composites subjected to clay-dominated soil environments. By leveraging their flexibility and capacity to capture nonlinear relationships, ANN models offer a robust framework for assessing durability-related risks.

1. **Effectiveness of ANNs:** The ANN models successfully predicted corrosion rates ranging from 0.014 to 3.60 mpy using three key input variables: sodium chloride concentration (0-4%), inhibitor dosage (0-5%), and exposure time (30-180 days). The best-performing model, FFBP, achieved a testing correlation coefficient of R = 0.998, MAPE = 30.43%, and RMSE = 0.071, outperforming traditional regression-based approaches.
2. **Development of a Generalized Model:** A generalized model for predicting corrosion rates using ANNs has been successfully developed, demonstrating low prediction errors and high correlation across all data subsets.
3. **Recommended Network Configuration:** The Feedforward Backpropagation (FFBP) network exhibited consistently better performance compared to the Cascadeforward Backpropagation (CFBP) model, which showed signs of overfitting during testing. The FFBP architecture is thus recommended for practical implementation.
4. **Sensitivity Analysis Insights:** Sensitivity analysis revealed that inhibitor dosage (DOI) is the most influential parameter, as removing it caused *R* to drop from 0.9985 to 0.7806. Sodium chloride concentration also significantly impacted model accuracy, while exposure time had a lesser but still notable effect. These results underscore the importance of including all input parameters to maintain model robustness.
5. **Dataset Scope and Limitations:** While the model demonstrated strong predictive performance, it was trained on a relatively small and uniform dataset (72 samples) from a single study. Future work should incorporate larger and more diverse datasets across varied environmental conditions to improve model scalability and field applicability.

## Data Availability

The datasets generated and/or analyzed during the current study are available from the corresponding author upon reasonable request.

## Abbreviations

AAD: Average Absolute Deviation
ANN: Artificial Neural Network
ANNs: Artificial Neural Networks
CFBP: Cascadeforward Backpropagation
FFBP: Feedforward Backpropagation
MAPE: Mean Absolute Percentage Error
MLP: Multilayer Perceptron
MSE: Mean Sum of Squares
R^2^: Determination Coefficient
R: Correlation Coefficient
RMSE: Root Mean Square Error
SVM: Support Vector Machine
STDV: Standard Deviation
